# Patient evaluation of a smartphone application for telehealth care of opioid use disorder

**DOI:** 10.1186/s13722-022-00331-4

**Published:** 2022-09-09

**Authors:** Jordon D. Bosse, Kim Hoffman, Katharina Wiest, P. Todd Korthuis, Ritwika Petluri, Kellie Pertl, Stephen A. Martin

**Affiliations:** 1 School of Nursing, Bouvé College of Health Sciences, Northeastern University, Boston, MA USA; 2 Institute for Health Equity and Social Justice Research, Northeastern University, Boston, MA USA; 3 OHSU–PSU School of Public Health, Oregon Health & Science University, Portland, OR USA; 4 Boulder Care, Portland, OR USA; 5 Addiction Medicine Section, Department of Medicine, School of Medicine, Oregon Health & Science University, Portland, OR USA; 6 Department of Family Medicine and Community Health, UMass Chan Medical School, Worcester, MA USA

**Keywords:** Opioid use disorder, Telehealth, Substance use disorder, Treatment for opioid use disorder

## Abstract

**Background:**

People with opioid use disorder (OUD) face barriers to entering and remaining in life-saving treatment (e.g., stigma, detrimental interactions with health care, and privacy concerns). Telehealth and related technology can reduce barriers to entering and staying in care. Patient feedback is critical to the development of these newer treatment approaches to ensure they are usable and do not inadvertently recreate treatment barriers.

**Purpose:**

Evaluate the perceived usability of existing and planned features of a mobile application (app) that facilitates delivery of OUD treatment via telehealth.

**Methods:**

People with current or prior experience with OUD treatment were eligible for the study. Participants (*n* = 31; 55% women) provided feedback on an interactive prototype demonstration via individual qualitative interviews and completed a quantitative survey on the app’s perceived usability. Descriptive statistics summarized the usability survey. We analyzed qualitative interview transcripts to elicit common themes.

**Results:**

Participants were primarily white (77%) with a mean age of 42.2 years (range 22–69). Participants rated the six major features of the current app as helpful (median response 5 out of 5) and appreciated the flexibility of conducting a visit from a place of their choosing. Participants regarded the five proposed components of the app, such as daily affirmations and medication treatment-related reminders (e.g., pick up medication at pharmacy, medication schedule), as useful features with medians 5 out of 5, and reported they would recommend the app to others for OUD care. Participant qualitative interviews provided additional information on perceived usability of existing and proposed app features.

**Conclusion:**

Our study suggests that an appealing, easy-to-use app—with tools and features that effectively support care—could circumvent existing barriers and foster sustained recovery.

**Supplementary Information:**

The online version contains supplementary material available at 10.1186/s13722-022-00331-4.

## Introduction

People with opioid use disorder (OUD) face extensive barriers to initiating and staying in treatment including fear of treatment, privacy concerns, time conflicts related to work and family obligations, poor treatment availability, and admission difficulty [1–3]. Limited availability of evidence-based medication treatment is also a significant barrier, especially among people who live in rural areas [4, 5]. Patients may have to travel long distances, join waitlists, or go without medication, increasing their risk of death by overdose. Other barriers to accessing and remaining in care include lack of transportation, competing responsibilities such as work and childcare, and pervasive stigma [6–8]. Telehealth may help people overcome some of these barriers to begin and stay in treatment.

For conditions such as OUD, anxiety, depression, or sleep disorders, telehealth achieves outcomes comparable to office-based care [9–14]. Pilot telehealth approaches for substance use disorders (SUDs) demonstrate effectiveness and high patient satisfaction [10, 13]. Prior to 2020, however, telehealth was rarely used for SUDs. If practiced, it was only to supplement in-person care rather than to deliver comprehensive treatment [15]. An analysis of over 400,000 commercially-insured patients treated for OUD between 2010 and 2017 determined that telehealth accounted for only 3 of every 1000 visits. Nearly all patients (99%) with telehealth appointments also had in-person visits; the modal number of telehealth appointments was a single visit [15]. The standard approach to telehealth often required a patient to travel to a clinic or office in order to take part in the visit, recreating existing barriers to treatment [14, 16].

The March 2020 suspension of a required in-person visit prior to prescription of controlled substances enabled buprenorphine treatment to begin via telehealth [14, 17–21]. Even so, most health care systems were understandably unprepared to abruptly provide telehealth for OUD. An analysis of 22 health centers early in the pandemic found only one center provided telehealth services directly to a patient’s home; all others required patients to go to a physical clinic to meet virtually [22]. As such, patients taking part in remote care continued to face physical barriers to entry. Clinicians refraining from telehealth amidst these relaxed regulations cited prescribing regulations as well as non-regulatory concerns [23]. The Department of Health and Human Services’ plan to make permanent the pandemic-suspended regulations may allay prescriber concerns and increase telehealth provision [24, 25]. Mobile phone applications are likely to be particularly useful for vulnerable populations; a recent study of 494 people who inject drugs found that most participants (77%) had a mobile phone that would allow telehealth care [26].

### User experience

Prior to the pandemic, the small scale of telehealth limited understanding of patients’ experiences with such services. Patient satisfaction and positive user experience correlate with better treatment retention and reduced mortality [27, 28]. A systematic review found telehealth to be associated with high patient satisfaction while being feasible and acceptable across a range of SUDs [29]. Many of the included studies utilized web browser-based telehealth treatments, and most were an adjunct to in-person care. Despite the World Health Organization’s recommendation to seek feedback from patients regarding their SUD treatment experience [30], patient input remains uncommon [31, 32] though growing as a result of implementation research.

Feedback from product users is more common in the technology design of fields other than health care. User centered design (UCD) is an approach in which people with the ‘problem’ the technology aims to address provide feedback at various stages of the development. UCD is critical to the development of a product that will be useful to and routinely used by the target population.

Usability (i.e., perceived usefulness and ease of use) is commonly evaluated through user centered design [33]. Perceived usefulness indicates the user of the product (application) believes it will enhance their experience [33]. In addition to being useful, technology must be perceived as easy to use, readily understood, adapt to different needs, and require minimal mental effort to deploy [33]. The current investigation follows recommendations that people who use drugs should be involved in design of services that serve them [34, 35].

### An app to facilitate care

**Fig. 1:**
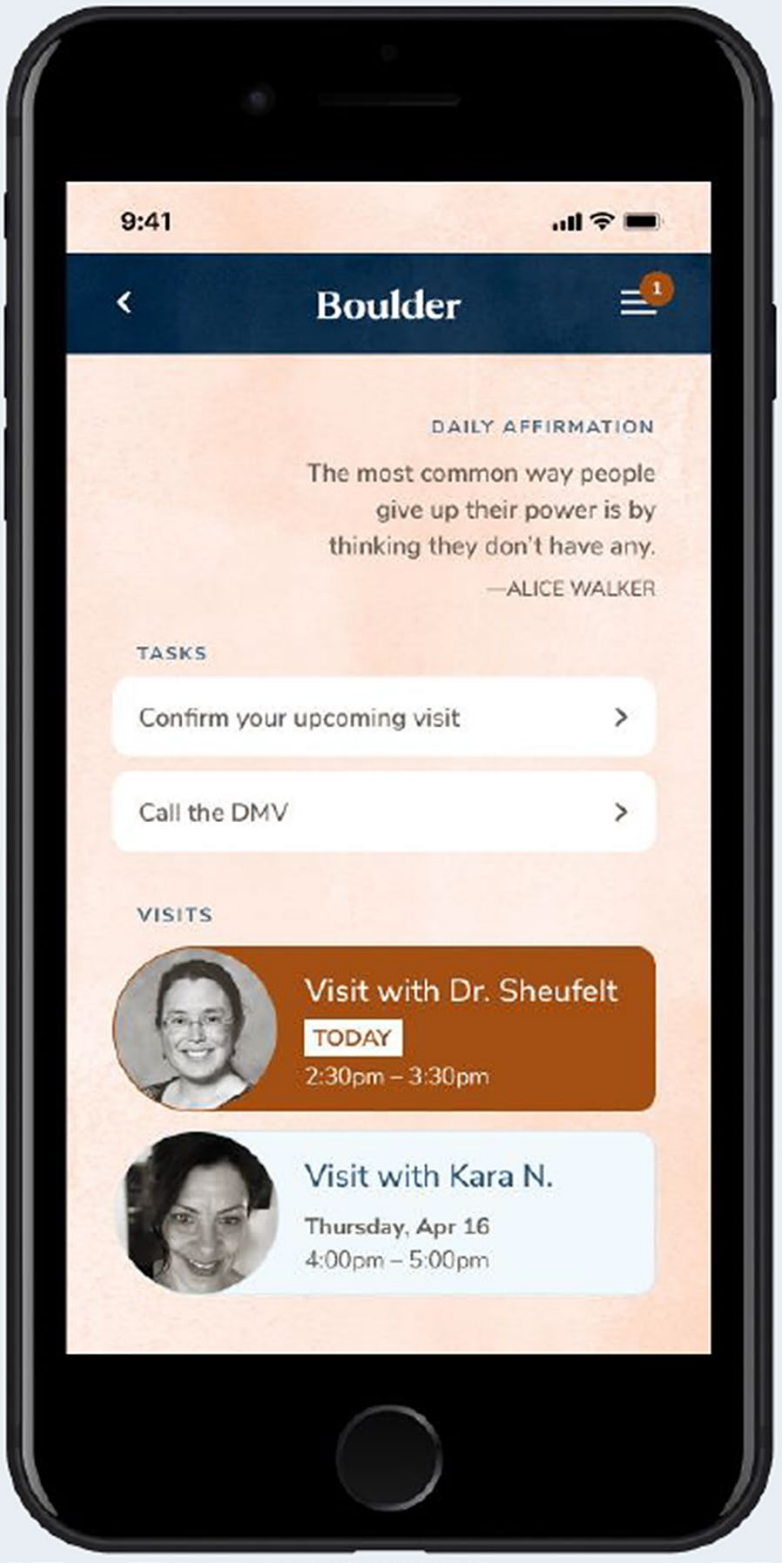
Example screen shot of application

Boulder Care was established in 2017 to provide patient-centered, telehealth-based OUD treatment. To facilitate effective care, the private company envisioned a smartphone app to facilitate patients’ longitudinal healing relationship with their Care Team. A National Institute on Drug Abuse (NIDA) Small Business Innovation Research (SBIR) award facilitated the development of an app to accomplish these goals [36]. This manuscript reports results from SBIR Phase I. Potential patients, clinicians, and software engineers co-developed a minimum viable product informed by best practices [37]. The initial patient-facing app scaffolded a complete telehealth experience, including secure, HIPAA-compliant text messaging with Care Team members, audiovisual visits, and an appointment calendar. The app was not available for the participants to download at this stage of product development, nor were they enrolled in any intervention or treatment because of the study. Participants were asked to provide feedback on a demonstration app based on screen shot examples. Figure 1 depicts a representative app screen displayed to participants during data collection. Demonstration of the app to potential users allowed them to identify both promising aspects and “pain points.”

To study potential user insights, Boulder Care collaborated with researchers at Oregon Health & Science University, leveraging the NIDA SBIR funding mechanism which encourages collaborations between small businesses and academic centers. The aims of this study were to obtain feedback from individuals with OUD and a history of (or current) treatment via in-person care. We wanted to learn from participants (1) the potential usefulness and ease of use of existing and proposed app features, and (2) potential additional features that would be helpful for individuals in treatment.

## Methods

### Eligibility

Individuals were eligible to participate if they were adults (21 years and older) who had ever been diagnosed with OUD and had either (1) a history of inpatient, residential, or outpatient OUD treatment or (2) were currently enrolled in treatment for at least three months. Participants were required to meet four additional eligibility criteria: (1) ability to read, speak, and understand English; (2) have access to wireless internet (Wi-Fi); (3) have an email address; and (4) have a mailing address to receive study compensation. Participants who took part in qualitative interviews needed a device (i.e., telephone, tablet, or laptop) to view screen shots of the application (such as Fig. 1) during video-based qualitative interviews.

### Recruitment

**Fig. 2:**
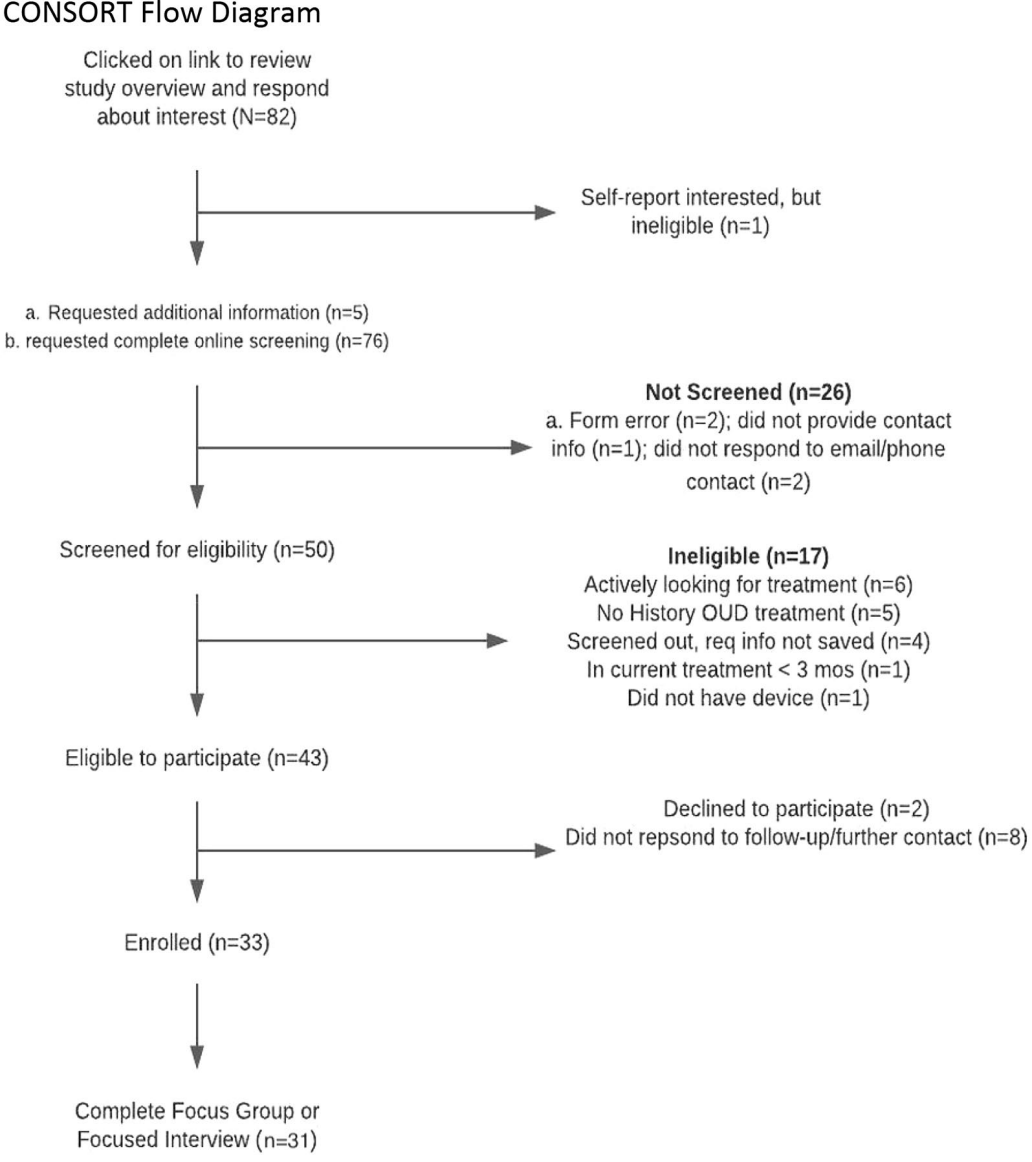
Participant screening and eligibility flow

We used paid social media ads (e.g., Twitter) to recruit a convenience sample [38] of 12 initial study participants. We recruited additional participants (n = 18) through distribution of study fliers in six primary care settings providing treatment for OUD, as well as by word of mouth from existing participants. Potential study participants (*n* = 53) completed an online screening (Additional file 1). Study staff followed up with eligible participants (*n* = 43) to answer questions and obtain informed consent (n = 31) (see Fig. 2). The remaining enrolled participants (n = 30) took part via Zoom (Zoom Video Communications Inc., San Jose, CA, USA) in a virtual interview or focus group using best practices for online qualitative data collection [38].

### Qualitative data collection and analysis

Participant interviews occurred either in small focus groups or as individuals (Additional file 2). Interviews were organized according to the convenience and schedules of the participants. Interview guides were semi-structured, allowing for further exploration into comments or concerns raised by participants [39]. During the interviews, participants were shown an interactive prototype, demonstrating both app content and function, and asked for their feedback. The demonstration included both existing app features (secure text chat and video visits) as well as proposed features (a daily task list, self-rescheduling, goal setting, guided meditation, and a video library). Digital audio recordings of the qualitative interviews were transcribed. A thematic analysis using a deductive coding scheme (derived from the topics of the quantitative analysis) identified respondent themes using the using the rigorous and accelerated data reduction (RADaR) technique for analyzing qualitative data. RADaR uses a team-based approach to coding and analyzing qualitative data [40]. Three investigators (KH, RP, KP) developed the coding scheme. Two coders (RP and KP) applied the codes to a subset of the interviews. This was reviewed by investigator KH who assisted the two coders in achieving consensus when there were uncertainties or applied codes that were discrepant with the coding scheme. After several rounds of iterative coding and review, the two coders coded the remaining transcripts.

### Quantitative data and analysis

Following the app demonstration, participants completed a web-based survey and rated the app’s features on a Likert scale. The Likert scales used in the assessments varied, one with a scale of 5 (1 to 5) and one with a scale of 11 (0 to 10) to better capture nuance in respondent choices. Five dimensions were assessed: Dimension 1 (Table 2) included *comfort with use*, *willingness to use the app for OUD treatment*, and *willingness to recommend the app*; Dimension 2 (Table 3) included *ease of navigation*, *app look and feel*, and *app fit with lifestyle*; Dimension 3 (Table 4) included *helpfulness of features*; Dimension 4 (Table 5) included *usefulness of features*; and Dimension 5 (Table 6) included the *likelihood of using potential features*. Parametric descriptive statistics examined response distributions.

Participants were compensated up to $80 for the time required to complete the study; compensation was in the form of reloadable Mastercard gift cards [41]. The Advarra Institutional Review Board reviewed and approved the study (Study Number 39405).

## Results

### Participant characteristics

Study participants (N = 31) were predominantly white (77%), with slightly more women (55%) than men (45%), and a mean age of 42.2 years (range: 22–69). Participants lived in five US states, with the majority (60%) living in urban and suburban settings (Table 1). Table 2 relates *participants’ satisfaction with their comfort using the app*, *willingness to use the app for OUD treatment*, and *willingness to recommend to others*; responses had a mean result of 8.5 or greater out of a possible 10 (11-point Likert scale, 0 to 10). The median level of comfort was 9 out of a possible 10. *Willingness to use* and *recommend the app* both had medians of 10 out of 10.

**Table 1:** Participant (N = 31) characteristics

	N	%
Age		
25–39	17	57
40–59	9	20
60+	4	13
Gender		
Women	17	55
Men	14	45
Race		
White	24	77
African American or Black	5	16
Another racial identity:	2	7
State of residence		
New Hampshire	10	32
Oregon	9	29
Ohio	6	19
Massachusetts	5	16
New York	1	3
Geography type		
Urban/suburban	18	60
Rural	12	40

**Table 2:** Dimension 1: comfort, interest and recommendation

Item	N^a^	Mean (SD)^b^	Median
How comfortable do you think you would be using the Boulder Care App?	25	8.9 (1.4)	9
If you needed treatment for OUD in the future, how interested would you be in receiving treatment over the Boulder Care App?	30	8.5 (6.3)	10
How likely would you be to recommend Boulder Care to a friend or family member who needed treatment for OUD?	29	9.0 (1.7)	10

Rated on an 11-point scale: 0 (not at all) to 10 (extremely)

^a^Ns vary as the survey did not require participants to answer each question

^b^Standard deviation

In Table 3, participants rated the app’s *usability* (*Mean* = 3.8), *appeal (Mean* = 3.9), and *lifestyle fit* (*Mean* = 4.1) on a 5-point Likert scale, 1 to 5. For each of the three items the median was 4 (Table 3). In Table 4, the ratings for the *helpfulness of the current six app* features had means ≥ 4.6 out of 5 while the median for each item was 5 (on a 9-point Likert scale, 1 to 5 with 0.5 intervals). Table 5 lists proposed features which were rated by a median of 5 (on an 11-point Likert scale, 0 to 5 with 0.5 intervals). Table 6 includes the *likelihood of acceptance of proposed app features* with medians of 4 to 5 and means ≥ 3.7 to 5 (on an 11-point Likert scale, 0 to 5 with 0.5 intervals).

**Table 3:** Usability, appeal, and lifestyle fit

Feature	N^a^	Mean (SD)^b^	Median
Ease of use	30	3.8 (0.9)	4
Appeal of app	29	3.9 (0.7)	4
Lifestyle fit of app	30	4.1 (0.9)	4

Rated on a five-point scale: 1 (not easy, not appealing, not a fit) to 5 (completely easy, completely appealing, fits completely)

^a^Ns vary as the survey did not require participants to answer each question

^b^Standard deviation

**Table 4:** Helpfulness of current app features

Item	N^a^	Mean (SD)^b^	Median
Appointments all in one place	31	4.6 (0.7)	5
Appointment reminders	31	4.6 (0.7)	5
Chat feature to message Care Team	31	4.9 (0.3)	5
Video chat feature to talk with Care Team	31	4.8 (0.6)	5
Virtual appointments from anywhere	31	4.7 (0.6)	5
Ability to cancel and reschedule using the app	30	4.6 (0.8)	5

Rated on a 9-point scale, 1–5 with 0.5 intervals: 1 (not useful) to 5 (completely useful)

^a^Ns vary as the survey did not require participants to answer each question

^b^Standard deviation

**Table 5:** Usefulness of proposed app features

Feature	N	Mean (SD)^a^	Median
Medication reminders	30	4.6 (0.8)	5
Complete labs reminders	30	4.5 (0.8)	5
Treatment goal reminders	30	4.5 (0.9)	5
Pick up medication from pharmacy reminder	30	4.6 (0.7)	5
Daily affirmations	30	4.8 (0.4)	5

Rated on an 11-point scale, 0–5 with 0.5 intervals: 0 (not useful) to 5 (completely useful)

^a^Standard deviation

**Table 6:** Likelihood of using proposed features

Feature	N^a^	Mean (SD)^b^	Median
A journal for recording thoughts and feelings	30	3.7 (1.3)	4
Prompts to write in journal	31	3.7 (1.3)	4
Interactive worksheets	30	4.1 (1.0)	4.5
Tracking days without use	30	4.5 (0.6)	4
Information to share with family and friends	25	4.4 (0.7)	4
Guided meditations	30	4.1 (1.1)	4.5
Sharing resources with other patients	27	4.4 (0.8)	5

Rated on an 11-point scale, 0–5 with 0.5 intervals: 0 (not useful) to 5 (completely useful)

^a^Ns vary as the survey did not require participants to answer each question

^b^Standard deviation

### Qualitative results

A deductive coding scheme identified three themes: (1) usability, appeal, and lifestyle fit; (2) helpfulness of current app features; and (3) usefulness of proposed features.

#### Theme 1: usability, appeal, and lifestyle fit

Respondents reported that the app was very appealing and fit their lifestyle. Individuals caring for family, working, or going to school expressed how the app would allow for balancing their many roles while attending to their treatment goals:

I think having the care coordinator, having the task list, reminders, whatever they are, for me, it would fit perfectly. I am a full-time mom, full-time employee, full-time student so utilizing the app it’s a quick check-in. We are on our phones every day. So I think it’s pretty feasible to get on the phone.

Another respondent echoed:

It would fit in perfectly for my life at least. For me, it would have covered everything that I would have going on in my life. Everything I would need.

Features designed to help track plans were seen as especially helpful:

I can be very distractible. I think it would help with accountability.

One respondent noted that the app was so appealing, she would like to see something similar available more widely:

I feel like they are making this awesome thing, why not make it accessible for other health care facilities to use?

#### Theme 2: helpfulness of current app features

Participants consistently noted that the app contained helpful features they would use during their treatment. An especially valued feature was the ability to schedule appointments with the app:

Definitely the scheduling and canceling of appointments would be the best for me. I have it in one place where my schedule is there and everything.

A respondent with previous experience receiving treatment at a busy clinic felt that having the app to connect with the clinic about appointments was an improvement over telephone calls:

That would be super helpful instead of having to get on the phone and do it on the phone because sometimes you can’t get through. Yeah. That would be helpful for those times. I think it makes it easy.

Participants universally appreciated the ability to refill prescriptions, chat with a trusted Care Team member, and avoid the hassle of driving to the clinic. For example:

It’s a great app, especially the—being able to get a test in the mail and get a prescription refill and having a trusting relationship with—not having to go through any of the crap of driving and checking in and run the lab—it’s great. It eases a million things. I mean, I wish it existed already.

Regarding appointments including video, respondents thought this service was important, especially during the pandemic. One respondent compared a video appointment with phone appointments:

I think it’s beneficial. That’s one of the aspects, especially right now [during the COVID-19 pandemic]. It’s just that contact with somebody; it does help when it’s not full [in-person] contact because it’s closer than just being over the phone.

#### Theme 3: usefulness of proposed features

When shown potential features for the app, respondents provided detailed feedback. Setting and tracking treatment or life goals was a preferred feature; three respondents highlighted potential benefits:

If I was using it, I think the goals is a great idea. I think that they—it would be more beneficial if they were worded as suggestions so as the app and as the provider is seeing that you are providing goals, you know, if you seem to be progressing well.

It’s useful because for many recovering addicts we don’t have anything to do in the beginning and we have ideas about we had that moment about going back to school, learning a trade of whatever and it’s set up already as part of my goals.

I think it’s great. I really do, it really is. Then you could also check back and it will make you feel the progress you made; you can actually see it. It’s you know, great.

Having a daily affirmation on the home screen was also a popular feature, though participants’ opinions varied about the content, preferring a degree of customization. Two respondents explained:

The wider the parameter and the more choice I have—this is just me. The more I feel like it’s kind of my app as opposed to someone trying to drive a message, if that makes sense.

Open this app every day and that’s how your start your day then to have a positive affirmation on there can really make a big difference.

One of the proposed features was the option of pre-loaded guided meditations to hear at their convenience. Respondents felt that this would be a useful optional tool, and some proposed additional audio options:

I would hope it would have a link you could click on to find something that you are interested in, music quiet, sounds, movements—exercises, stretches…

Finally, medication reminders were seen as a potentially helpful feature:

We need to be reminded sometimes. Getting some structure is really good. You know, being accountable, getting back to being responsible, taking your medication.

### Follow-up survey responses

In addition to numerical ratings of the app’s perceived usefulness, ease of use, helpfulness, and life fit, some participants responded to open-ended questions and provided suggestions for improvement. Participants also provided open-ended responses at various points in the survey; for example, regarding the reason for their willingness to recommend the app to others and ideas for educational content and additional app features that would be beneficial.

#### Strengths of app

In their survey responses, participants mentioned how this app could reach individuals who might otherwise feel uncomfortable coming into a clinic for treatment (e.g., due to social anxiety, stigma, seeing others they might know). One respondent wrote:

It’s a new innovative way to get confidential treatment. Makes things less scary and less daunting. Sometimes calling places is hard and having the ability to get connected through an app eases that pressure.

#### Specific app features

Participants also rated *ease of use*, *appeal*, and *lifestyle fit* high (Table 3). All current features of the app (Table 4) and proposed app features (Table 5) were deemed highly helpful and useful. Among additional proposed features, the ability to track days without using a substance or substances, experience guided meditations, and engage with interactive worksheets were reported to have a higher likelihood of being used (Table 6).

#### Challenges and concerns

Among the few respondents who indicated on the follow-up survey that they were not likely to recommend the app, reasons for this response included concerns about technology (e.g., “what if the Internet goes down?”) and preference for in-person visits. One respondent wrote,

In person accountability and connection … has always been an important piece of my recovery. It’s hard to form that supportive attachment through a computer monitor.

A few participants suggested telehealth might not be for everyone and had specific concerns regarding people early in recovery. One person wrote,

As a person with lived experience [with OUD treatment] I feel like I would be most likely to recommend the app to someone if they had a reasonably stable life already, I feel like extreme cases may require more support than the app can initially provide.

#### Educational content

Most (66.7%) respondents were interested in educational content via multiple methods, including written/downloadable documents, images or graphics, or videos either on a website or in the app. The proposed video education library was seen as positive, with nearly two thirds (60%) indicating interest in learning through videos. There were differing opinions about whether they should be a required part of treatment. For example, one respondent noted: “If we are not required to complete a specific number of videos or modules per week, I would use occasionally.” For those who *would* watch videos, they maximum amount of time they wanted to spend ranged from 5 to 25 min. Videos delivered by others with lived experience with OUD about their challenges and triumphs were of strong interest.

#### Additional recommended features

Participants offered a set of suggestions to improve the app’s support for people in recovery: integrating appointments in the app with other apps such as calendars; providing resources for in-person recovery groups and activities (e.g., the app could locate nearby peer support meetings based on their location); and identifying local resources to assist with social needs (e.g., food, housing, government ID).

## Discussion

Participants found the interactive app prototype simple to use, appealing, and useful in overcoming barriers typically associated with treatment. They noted that the app provided convenient features, specifically re-scheduling appointments, receiving notifications regarding their care, and interacting with their Care Team from wherever they were comfortable and have cell or Wi-Fi reception. The convenience of communicating via secured chat has also been identified in another study of patient perceptions [42]. Participants in another study reported the ability to communicate with a provider via secure message was quicker, easier, more direct, and made the Care Team easier to access—elements that support retention in care [24].

Certain limitations of telehealth should be considered, however, such as lack of a convenient private setting to carry out videos and technology challenges such as antiquated devices. Despite these challenges, respondents supported the app’s ability to improve interaction with the Care Team. Participants appreciated the app’s ability to collaborate regarding tasks and goals related to their recovery. In a study of similar size and scope, goal-setting features were routinely used by a third of the sample [24]. Flexible, individualized treatment approaches are more likely to increase treatment [43, 44].

The time demands and opportunity costs of in-person care—transportation, work, childcare, money—all impact treatment continuity. Participants supported the app’s ability to overcome these common barriers and vulnerabilities in the cascade of care. In addition, the interactive app prototype allows patients to readily communicate with their Care Team and access specific education, both of which can support retention [24, 44]. A few participants expressed preference for in-person visits, noting a better “connection” with a clinician, and these individual preferences should be honored. Depending on patient preferences, a therapeutic connection can be successfully achieved via telehealth [43–46]. As telehealth options become more available, people may become more comfortable with this format [27].

Participants’ recommendations for additional features centered on building connections with others with lived experience, either through the app or in their own communities. Peer recovery specialists provide unique support rooted in their life history as well as informational and instrumental support [47]. These specialists have been found by multiple studies to positively impact patients’ lives, including during the COVID-19 pandemic [48, 49]. Participants also recommended the app provide a list of local resources to meet social needs, as they often face unemployment, food insecurity, and unstable housing [50].

### Limitations and strengths

Nearly half of our participants’ feedback came through individual interviews—rather than focus groups—due to the limited time period of the SBIR Phase I study. Discussing feedback in a group setting can offer constructive juxtaposition of one’s thoughts with those of other participants [39]. Utilizing Zoom to meet with participants, however, allowed us to learn from participants in different areas of the US, in contrast to the location restriction imposed by in-person groups.

Our study was limited as participants were a convenience sample and not necessarily representative of patient demographics in terms of opioids used, duration of use, treatment attempts, geography, and demographics. For example, participants were mainly White. Additionally, for remuneration purposes, respondents were required to have a mailing address, which may have excluded participants with unstable housing. More than two thirds (68%) of the sample are from states whose rate of opioid overdose per 100,000 was well above the US average (15.5) in 2019: Ohio (31.5), New Hampshire (29.1), and Massachusetts (28.9) [51]. This prevalence may alter participants’ perspectives in a way that is limiting. A study strength is that our sample includes women [52] and a substantial number of people (40%) residing in more rural areas, both of whom are generally underrepresented in OUD-related research. Future studies should explore the app usability among diverse groups (e.g., racial and ethnic groups, sexual and gender-diverse individuals, and non-English speakers) as well as test the app with individuals who do not have prior experience with OUD treatment. Our small sample size also limited statistical power and the ability to conduct more detailed analyses or between group comparisons (e.g., barriers for urban vs. rural-dwelling participants). Despite these limitations, our results suggest that telehealth for OUD is a promising way to expand access to care.

## Conclusion

People seeking treatment for OUD face many barriers to entry, and those who access care may have difficulty continuing. While some of these barriers can be addressed through low-threshold, harm reduction principles of care, additional challenges related to the physical setting can be addressed by telehealth. During and after the COVID-19 and OUD syndemic, a highly accessible, evidence-based, reliable treatment solution is critical. Our findings begin to characterize patient preferences for a related app. Participants highlighted numerous barriers to entering and continuing treatment (such as stigma, attending to family obligations, and employment) that would be removed by their participating in comprehensive telehealth care. This app technology fits into individuals’ daily lives by supporting day-to-day tasks, needs, and goals and has the potential to reduce or eliminate challenging treatment barriers. Patient input into the design of treatment apps is essential for developing interventions that improve treatment experience and patient-centered outcomes [53]. Our study suggests that an appealing, easy-to-use app—with tools and features that effectively support care—could circumvent existing barriers and foster sustained recovery.

## Supplementary Information

**Additional file 1.** Eligibility screening (phone-based).

**Additional file 2.** Focus group guide.

## Data Availability

The datasets during and/or analyzed during the current study available from the corresponding author on reasonable request.

## Abbreviations

NIDA: National Agency on Drug Abuse
OUD: Opioid use disorder
RADaR: Rigorous and accelerated data reduction
SBIR: Small Business Innovation Research
SUD: Substance use disorder
UCD: User-centered design
