# Supplementary material for: Patient evaluation of a smartphone application for telehealth care of opioid use disorder
Source: Addict Sci Clin Pract. 2022 Sep 9;17:50. doi: 10.1186/s13722-022-00331-4 (PMC9462609; doi:10.1186/s13722-022-00331-4)
Supplement: Supplementary file 1 — Additional file 1. Eligibility screening (phone-based). [file 13722_2022_331_MOESM1_ESM.docx]

**Eligibility Screening (Phone-Based)**

1. How old are you as of today? _____ [if <21, exclude]

2. Have you ever been diagnosed with opioid use disorder (OUD)? Yes I’m not sure No [exclude]

a. Are you currently in treatment for OUD? Yes No

i. If YES: How long have you been in your current treatment?

(if <1 month, please enter “0”) _____ months [< 3 months = exclude]

ii. If YES: Does your current treatment include medication? Yes No

iii. If NO: Do you currently need treatment for OUD? Yes [exclude] No

b. Have you ever received treatment for OUD? Yes No [exclude]

3. Did your treatment ever include medication? Yes No

a. Which medication(s)?

[buprenorphine (Subutex); Buprenorphine/naloxone (Suboxone); methadone; naltrexone

injection (Vivitrol); another medication (please describe: ____________________)

[exclude if no history of buprenorphine or buprenorphine/naloxone]

4. Do you have regular access to a smart phone? Yes No [exclude]

5. Do you have regular access to wireless internet? Yes No [exclude]

6. Do you have a place to receive mail? Yes No [exclude]

7. Do you have access to a computer or tablet to take part in an online meeting?

Yes No [exclude]
