# Supplementary material for: Patient evaluation of a smartphone application for telehealth care of opioid use disorder
Source: Addict Sci Clin Pract. 2022 Sep 9;17:50. doi: 10.1186/s13722-022-00331-4 (PMC9462609; doi:10.1186/s13722-022-00331-4)
Supplement: Supplementary file 2 — Additional file 2. Focus group guide. [file 13722_2022_331_MOESM2_ESM.docx]

**Focus Group Guide**

[Slide 1] Welcome page

[Slide 2] When you open the app for the first time, you get a bit of information about Boulder and what we think makes our program different from others.

[Slide 3] One of the main differences is being able to do almost anything related to care from home or some other private place.

[Slide 4] We know from the science that medication works for people so that’s a big part of our program.

[Slide 5] We hope that by having fewer barriers to treatment that it will be able to be just part of the usual routine.

[Slide 6] This is the sign in screen

[slide 7] This is the home screen. Let’s start at the top. One of the things we want to do in the future but haven’t done yet is to include a daily affirmation at the top of the home screen.

What do you think about including a daily affirmation? What would you like about it? What would you not like about it? What kinds of things should the messages say/not say?

You can see the task list for today. You can see here (check mark next to “Take my buprenorphine”) that this task is complete.

How helpful would you find having a to-do list like this? What tasks would it be helpful to be able to keep track of here?

Then, you can see the person has a few appointments scheduled. The visit highlighted in green is scheduled to start in 5 minutes. Joining a visit is easy—you just click ‘join’. [slide 8] The video visit opens, and you can see the care team member you’re working with. This happens to be Ginger, a terrific peer coach.

What concerns might you have about seeing someone on a video like this instead of in person? As you got started, what would you want to know about them or about the visit in general?

[Slide 9] You can see here that we have a task added—Watch a Video: Recovery Goals. Clicking on the task will take us to the next screen [Slide 10] and you would watch the video

How likely would you be to watch a video on a recovery theme? What would make you more likely to watch it? Less likely to watch it? Who would you want to be delivering the information in the video? How long would you be willing to watch a video? What about other formats—text articles? Audio only/podcast? (how likely to read/listen? What would help? How long willing to spend?)

You can see that the top video is grayed out, meaning you’ve already watched it. Would you want to be able to watch videos more than once?

(One of the thoughts we had was that you could watch a short video, say, while waiting for your medication do dissolve. What do you think about that?)

[Slide 11] Then, when we click back to the main screen, you can see the video task has been deleted.

Now, let’s say I look at the next appointment and realize I won’t be available . I can click on these 3 dots, and that will give me the option to cancel or reschedule my appointment.

[Slide 12] This is another feature that is in the works—self-scheduling. You can see the idea here is that you would be able to pick the day and see the times your provider is available.

[Slide 12, cont.] You can see here that a warning came up to let us know that ….. and then you have the option to cancel and go back to the schedule to find another day. Or you can choose to book anyway.

[slide 13] The calendar visit was updated here to reflect the new appointment

[slide 14-16] From the home screen, you would go to the main menu to reach out to your care team. In this instance, we’d want to reach out to the Care Advocate to let them know that you had to reschedule your visit and might run out of medication before the next one.

You can see the response from the Care Advocate after they talked to the provider about the need, they the provider is going to submit a refill, but wants a lab test first.

[slide 18] Now when we go back to the home screen, we can see that there are new to-do items on the list, including picking up medication and completing the home lab test

How helpful would it be to have a task to remind you to pick up your medication?

[slide 19-22] then the app walks you through taking the test. Once it’s packaged, you would drop it in the mail.

What do think about doing labs this way? What might make it difficult for you to do this or what barriers might there be? What questions or concerns would you have? What do you think you would like about it?

How much time do you think it would take you to get the sample in the mail once you’ve packaged it up? What could make it take longer to get the sample to the mail?

[slide 23-27] Then we can see here that the test is marked completed, and we have a notification, which here indicates that that there’s a new message from Ginger. When we return to the home screen [slide 28] “meditate for 10 minutes” has been added to the task list.

Have you used meditation in the past? If not, would you be willing to try it? What would you want to happen when you click on the ‘meditate for 10 minutes’ task? (e.g. bring to information about meditation, bring to an audio file/guided meditation? Start a timer?)

What other strategies have you used when you had trouble sleeping? What other suggestions could a peer coach provide? (for those strategies…what could we build into the app to support those coping skills?)

You will also notice that there’s a task “Register for classes” listed here. When we click on that task, you get brought to a screen [slide 29] that shows that you have set a goal to start taking college classes. You can see here that the first 3 steps toward achieving your goal have been marked as complete.

What do you think about having a goal setting feature? What kinds of goals would be important for you to track? How would you want to track them? Would you want the individual steps to show in your daily to-do list? How would you see yourself using this feature? (e.g. to have discussion with provider/care team? On regular basis?) How else would you want to track progress toward your goals? There’s a progress bar at the top, it shows you’re about halfway there. How helpful is the progress bar? Is there anything you would want to add to it or change about it?

Would you want to receive reminders or notifications about your tasks to reach your goals? Or other general tasks (e.g. pick up medication)? What kind of notifications are helpful (in app only? Push notifications? Etc.) How often would you want to get notifications?

In thinking about this particular goal—goal setting-- Often we encourage goal setting to be completed with the peer coach. Would you want to enter your goal and the steps to get there yourself or have the peer coach enter the goals/steps after having a discussion with you? Would it be helpful to have a set of goals to choose from or would you want to be able to enter everything with free text?

What would you like to see/have happen after you completed all of the steps toward this goal?

[slide 21-24] Then we can see here that the test is marked completed, and we have a notification, which here indicates that that there’s a new message from Ginger.

That concludes the demo.

1. What did you think of the app and features overall?
2. How do you think the Boulder app would fit into your life?
   1. Follow-up as needed: what specifically about X…; what would make it fit into your life better?]
3. Of the features we just reviewed, which would you find the most helpful?
   1. Follow-up as needed: What specifically about X would be helpful?]
4. What feature(s) would not be helpful?
   1. Follow-up as needed: What specifically about X would not be helpful?]
5. If you could change one thing, what would that be?
6. What other features could we add/include to improve the app?
   1. Then, looking at our list [review list], if we could only add of these, which would you choose?
7. One piece of our program we’re working on building is education about substance use, treatment, and recovery. What topics and messages do you think would be important to include?
   1. For specific topics (e.g. ‘medication’—what, specifically, do people need to know?)

Probing questions to be included as necessary/appropriate:

- Did anyone else have a similar/different experience?
- Does anyone have a different idea/opinion?

Let’s wind down our conversation in the next few minutes. [review/summarize key points group has shared] What other feedback do you have about what you have seen tonight?

**Close**

Thanks, again, for your time and feedback in this conversation. We really appreciate it.
